# Facile method for enhancing the CO_2_ adsorption capacity of zeolites through vacuum-assisted alkaline treatment†

**DOI:** 10.1039/d5ra01559f

**Published:** 2025-06-11

**Authors:** Lihong Shui, Junjie Zhou, Yunan Wang, Yichao Lin

**Affiliations:** a Zhejiang Key Laboratory of Advanced Fuel Cells and Electrolyzers Technology, Ningbo Institute of Materials Technology and Engineering, Chinese Academy of Sciences Ningbo Zhejiang 315201 P.R. China wangyunan@nimte.ac.cn yclin@nimte.ac.cn; b School of Chemistry and Chemical Engineering, Harbin Institute of Technology Harbin 150001 P.R. China; c University of Chinese Academy of Sciences Beijing 100049 P.R. China

## Abstract

Zeolites are considered as promising CO_2_ adsorbents due to their affordability, exceptional stability, and porous characteristics, yet they are still facing the challenges of low adsorption capacity and selectivity. In this study, we present a straightforward method to significantly improve the CO_2_ adsorption performance of zeolites through vacuum-assisted alkaline treatment. Under alkaline conditions, the pore structure and surface functionality can be modulated through desilication, dealumination, and ion exchange. Additionally, vacuum conditions aid in releasing gas molecules trapped in the pores, facilitating the solution entry. Three typical zeolites, including SSZ-13 (CHA), T (ERI/OFF) and NaA (LTA) zeolites, are utilized through the vacuum-assisted alkaline treatment. At an optimized pH of 12, their CO_2_ adsorption capacities increase by 7.67% to 16.99%. The significant enhancement in the CO_2_ adsorption capacity is attributed to the modified pore size and pore volume. Thus, we suggest that the vacuum-assisted alkaline treatment is an effective approach for improving gas adsorption performance.

## Abstract 2

A straightforward and universal method is presented to significantly improve the CO_2_ adsorption performance of zeolites through vacuum-assisted alkaline treatment.

## Graphical abstract

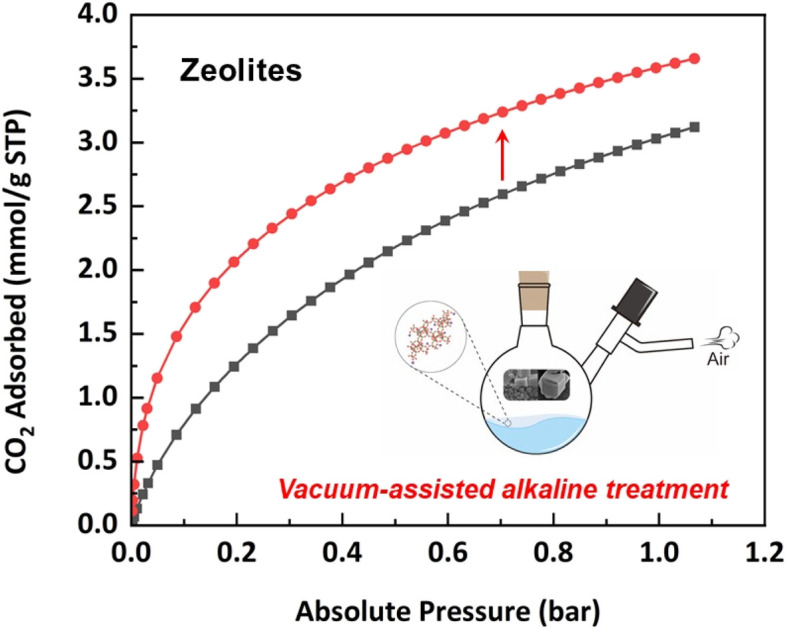

## 1. Introduction

The issue of global warming has led to a global consensus on the imperative to decrease CO_2_ emissions. By far, the majority of anthropogenic CO_2_ emissions originate from the burning of fossil fuels such as gasoline, natural gas, and coal. Notably, coal-fired power plants alone contribute to approximately 30% of the total CO_2_ emissions, drawing substantial attention from both the scientific community and industrial sectors.^1,2^ However, there is a widespread reliance on coal-fired power, constituting a significant share of the global energy production landscape. Alongside the progression of renewable energy sources, the incorporation of carbon capture, utilization, and storage (CCUS) technologies within coal-fired power plants presents a promising and efficient approach to curbing CO_2_ emissions. Diverse porous materials, including metal–organic frameworks,^3^ carbons,^4^ and zeolites,^5^ have been investigated for CO_2_ capture. Among these materials, zeolites have demonstrated exceptional proficiency as CO_2_ adsorbents, rendering them highly attractive for large-scale industrial CO_2_ capture applications due to their durable structures and cost-effective manufacturing techniques.

Zeolites constitute a category of inorganic porous aluminosilicates with adjustable pores and high surface areas.^6–8^ Substituting silicon for aluminum in the framework introduces a negative charge, necessitating the use of cations for charge balance. These counter ions play a crucial role in determining both the pore dimensions within the framework and the gas adsorption capacity of zeolites. For example, substituting Na^+^ in zeolite Na-A (LTA) with higher-valence Ca^2+^ leads to an increase in pore size from 4 Å to 5 Å, enhancing the CO_2_ adsorption capacity by enabling better access to the internal pores.^9^ In contrast, the replacement of Na^+^ with the larger K^+^ results in the contraction of LTA pore size to 3 Å, which is smaller than the diameter of a CO_2_ molecule (3.3 Å), causing minimal CO_2_ adsorption.^10,11^ In principle, an increase in cation size enhances the basicity of the adsorption site, facilitating the adsorption of acidic molecules such as CO_2_.^12^ Nevertheless, smaller cations reduce the spatial constraints, thereby improving accessibility to the internal space of zeolites. In essence, the introduction of cations can alter the adsorption properties by tailoring the acidity, pore structure, window size and internal electrical field.^13,14^

CO_2_ adsorption capacity of zeolites is significantly influenced by the distribution of cations within the framework, which is dependent on the efficacy of cation-exchange engineering. Cation exchange in zeolites has been commonly achieved by immersing desired cations in a stirred solution at an elevated temperature in the presence of air.^15,16^ However, the small pore size of zeolites presents a challenge for the impregnation or diffusion of cations into the innermost pores, as these pores are usually occupied by guest molecules. Herein, cation exchange is impeded within the inner part of zeolites, impacting CO_2_ adsorption negatively.

In this study, we propose a vacuum-assisted cation-exchange strategy to enhance cation exchange in zeolites. Previous studies have indicated that alkaline cations can improve CO_2_ adsorption, and treating zeolites with sodium hydroxide (NaOH) solution can further modify the pore structure through partial desilication.^17,18^ Our improved approach is to treat the zeolite with a NaOH solution under vacuum conditions to promote diffusion of guest molecules from the internal pores to the exterior. Three kinds of zeolites with various topologies, including SSZ-13, T, and LTA have been treated to demonstrate the efficacy and applicability. SSZ-13, T, and LTA zeolites were selected because they are commercially available, possess distinct and representative structural topologies, and exhibit high CO_2_ adsorption capacities. All these modified zeolites exhibit substantial enhancements in CO_2_ adsorption, mainly attributed to the alterations in pore size and pore volume.

## 2. Experimental

### 2.1 Vacuum-assisted alkaline treatment

Sodium hydroxide (NaOH) solutions with a pH range of 11–14 were initially prepared for subsequent use. A 40 ml alkaline solution was poured into a 50 ml one-neck flask, followed by the addition of 0.5 g of zeolite under stirring. The system was then evacuated using an oil pump until no more bubbles were emitted, creating a vacuum environment. The stirring continued for 24 hours thereafter. The resulting product was later centrifuged and dried at 80 °C. All zeolites utilized in the experiment were commercially available and left untreated. The resulting samples were labeled as Sample-V or A-pH_*n*_, where “Sample” refers to the zeolite name, V or A denotes the vacuum or atmospheric conditions, and pH_*n*_ represents the pH level of the NaOH solution used. For comparison, control samples were prepared using a conventional alkaline treatment in air without applying a vacuum condition. The control samples were labeled as Sample-pH_*n*_.

### 2.2 Characterizations

The surface micromorphology of the samples was examined using a field-emission scanning electron microscope (FE-SEM, Hitachi S-4800). X-ray powder diffraction (XRD) analysis was conducted on a Bruker AXS D8 Advance diffractometer utilizing Cu Kα radiation at ambient temperature. The 77 K nitrogen adsorption/desorption isotherms and CO_2_ adsorption isotherms were measured using the ASAP 2020 M apparatus from Micromeritics. Prior to adsorption testing, the samples underwent an 8-hour treatment at 160 °C to eliminate any adsorbed molecules in the pores. X-ray fluorescence spectrometer (XRF) test was performed using a Bruker S8 TIGER instrument (Germany). Inductively coupled plasma optical emission spectrometry (ICP-OES) measurements were carried outs using a SPECTRO ARCOS instrument (Germany). Before testing, the samples underwent room-temperature acid digestion, with a nitric acid to hydrofluoric acid ratio of 3 : 1.

## 3. Results and discussion

### 3.1 Structural and morphological characterizations

**Fig. 1:**
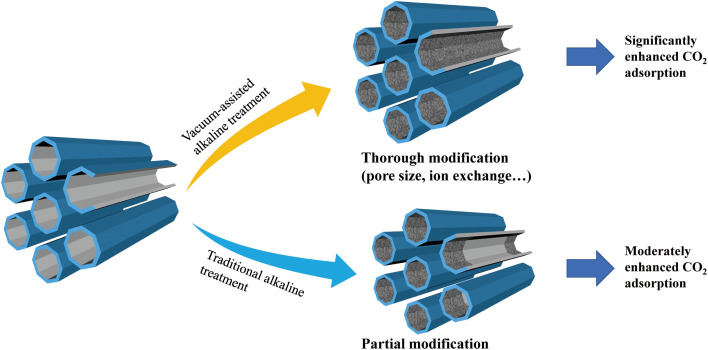
Benefits of vacuum-assisted alkaline treatment.

**Fig. 2:**
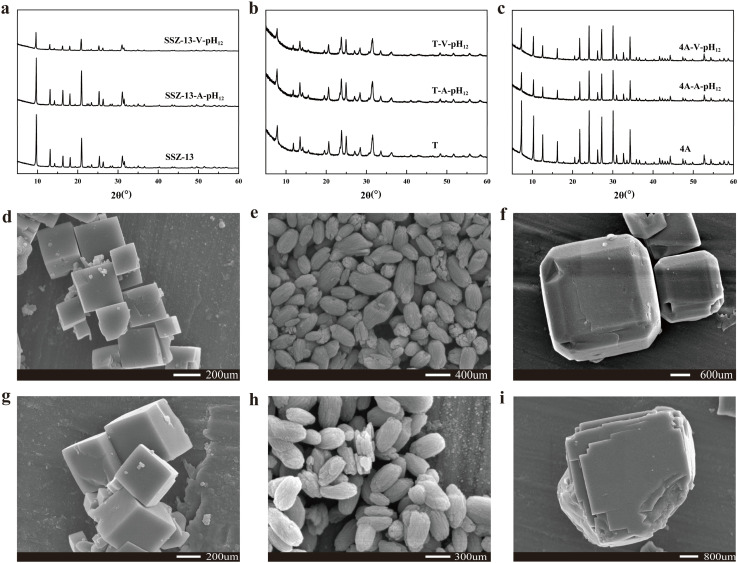
Structure and morphology characterizations of zeolites before and after vacuum-assisted alkaline treatment. (a–c) XRD patterns of SSZ-13 (a), T (b) and NaA (c) zeolites; (d and g) SEM images of SSZ-13 before (d) and after (g) vacuum-assisted alkaline treatment; (e and h) SEM images of T zeolite before (e) and after (h) vacuum-assisted alkaline treatment; (f and i) SEM images of NaA before (f) and after (i) vacuum-assisted alkaline treatment.

Alkaline treatment is commonly used to modify the pore surface/structure of zeolites. However, the small pores of zeolites are typically occupied by guest gas molecules adsorbed from air, hindering the diffusion of alkaline solution into the innermost pores. As a result, ambient-pressure alkaline treatment primarily modifies only the superficial pores. In this study, we conducted alkaline treatment under vacuum conditions using a vacuum pump to evacuate the system. Under vacuum, the trapped gas molecules inside the zeolite pores readily diffuse out, significantly enhancing the penetration of the alkaline solution into the inner pores (Fig. 1). This approach aligns with the work of Xu *et al.*,^17^ who proposed a similar vacuum-assisted strategy for pore repair in NaA zeolite membranes using sodium alginate and CaCl_2_ solutions. X-ray diffraction (XRD) analyses were conducted to evaluate the structural changes in the zeolites (SSZ-13, NaA, and T zeolite) before and after alkaline or vacuum-assisted alkaline treatment (Fig. 2a–c). The XRD profiles of the pristine SSZ-13, NaA, and T zeolites align with the standard patterns, with no additional peaks observed, indicating the high purity of the zeolites. One can see that there are no discernible alterations of the XRD patterns for the zeolites after alkaline or vacuum-assisted alkaline treatment, indicating the well preservation of the crystal structures. Furthermore, the microscopic morphologies of SSZ-13, NaA, and T zeolite were also probed through scanning electron microscopy (SEM) images (Fig. 2d–i). The SSZ-13 particles exhibit a cubic morphology with a diameter of approximately 300 nm. NaA particles display a spindle-shaped morphology, measuring about 350 nm in length. T zeolite particles show a near-cubic morphology with a diameter of approximately 2 μm. It shows that the morphologies of three zeolites remain unchanged after the treatment as well. Therefore, the alkaline or vacuum-assisted alkaline treatment does not affect the particle integrity or disrupt the crystal structure of the zeolites.^18–20^

### 3.2 Pore size analysis

**Fig. 3:**
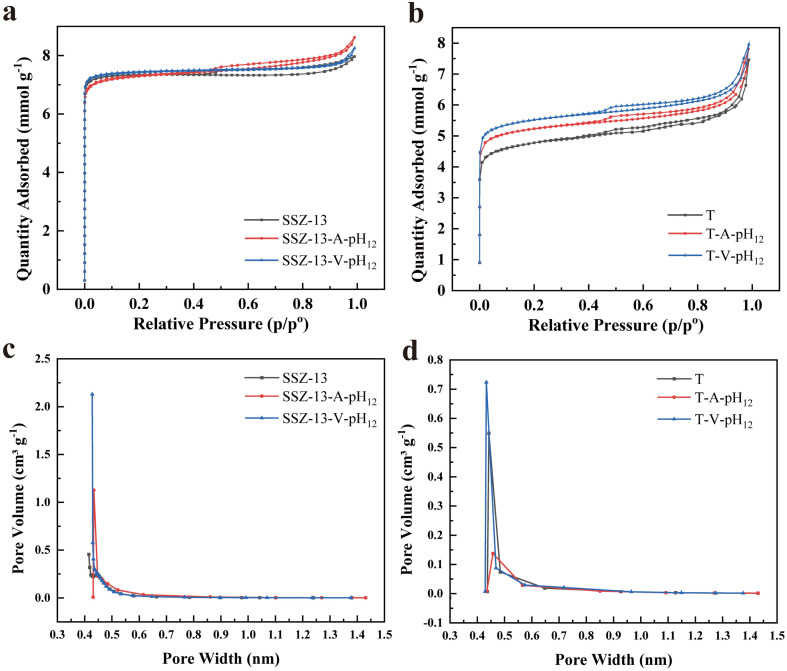
Pore structure characterizations. (a and b) 77 K N_2_ adsorption/desorption isotherms SSZ-13 (a) and T (b) before and after alkaline treatment with or without vacuum condition. (c and d) H–K pore size distribution curves of SSZ-13 (c) and T (d) zeolites before and after alkaline treatment with or without vacuum condition.

The pore sizes of the as-prepared samples were investigated by 77 K N_2_ adsorption/desorption measurements. Due to the limited pore size of NaA that hinders the penetration of N_2_ molecule, we focused on SSZ-13 and T zeolites specifically. Nevertheless, the pore size variation of NaA upon the vacuum-assisted treatment can be estimated based on the findings of SSZ-13 and T zeolites. Fig. 3a and b depict the N_2_ adsorption/desorption isotherms at 77 K for SSZ-13 and T zeolites, respectively. According to the International Union of Pure and Applied Chemistry (IUPAC) classification, both SSZ-13 and T zeolites exhibit characteristic features of type I and IV isotherms, indicating their possession of both microporous and mesoporous structures.^21^ It is well known that SSZ-13 and T zeolites possess crystalline microporous structures. The presence of mesopores in the commercial zeolites can be attributed to the structural defects that facilitate the migration of guest molecules through the inner pores.

In Fig. S1 and S2,† we compare the N_2_ adsorption/desorption isotherms of SSZ-13 at 77 K that are treated under different pH conditions. Compared with the commercial zeolite, treatment with neutral water does not change the N_2_ uptake apparently, whereas the capacity of N_2_ adsorption decreases with the treatment of pH = 11. We posit that Na^+^ with the larger ion diameter replaces H^+^ in the framework, leading to a contraction in pore size and the resulted uptake reduction.^22,23^ Under higher pH, Na^+^ exchange is expected to be more pronounced, but on the other hand, elevated pH condition facilitates desilication and dealumination, introducing imperfections and enlarged pore volume. N_2_ loading increases to a maximum when treating zeolite at pH = 12. Under even higher pH, such as pH = 13, an excessively alkaline environment can trigger extensive desilication and dealumination, potentially causing framework collapse and structure distortion.^13^ As a result, a substantial decrease in N_2_ adsorption and a noticeable decline in specific surface area are observed. Considering the effects of both Na^+^ exchange and desilication/dealumination, the optimal pH value of the vacuum-assisted treatment for CO_2_ adsorption is 12.

Subsequently, we focus on analyzing the pore structure of SSZ-13 treated at pH = 12, designated as SSZ-13-V-pH_12_. For comparison, SSZ-13 was also processed using alkaline treatment under ambient pressure, labeled as SSZ-13-A-pH_12_. Both SSZ-13-V-pH_12_ and SSZ-13-A-pH_12_ demonstrated slightly enhanced BET surface areas compared to the pristine SSZ-13 (Fig. 3a and Table 1). The micropore sizes were analyzed by using the Horvath–Kawazoe (H–K) method. As shown in Fig. 3c, all samples display a narrow distribution of micropore sizes ranging from 0.4 to 0.5 nm. The corresponding micropore volumes follow the order: SSZ-13-V-pH_12_ > SSZ-13-A-pH_12_ > SSZ-13, indicating that the vacuum-assisted alkaline treatment contributes to an increase in the micropore volume of SSZ-13. In addition, comparing the pore size distribution of SSZ-13 treated at different pH values reveals a consistent trend. As shown in Fig. S2,† this trend underscores the great contribution of the vacuum condition to augment the microporous volume. Fig. 3d presents the H–K pore size distribution of T-type zeolites before and after alkaline treatment, with or without vacuum conditions. Similar to the results of SSZ-13, the microporous volume of T-V-pH_12_ was significantly larger than that of pristine T zeolite and T-A-pH_12_.

**Table 1:** Textural properties and CO_2_ adsorption capacities of zeolites

Sample	*S*_BET_ a (m^2^ g^−1^)	*V*_total_ b (cm^3^ g^−1^)	*V*_mic_ c (cm^3^ g^−1^)	CO_2_ uptake^d^ (mmol g^−1^)	
				0.15 bar	1 bar
SSZ-13	488.93	0.28	0.25	1.05	3.12
SSZ-13-V-pH_11_	473.29	0.29	0.23	0.87	2.58
SSZ-13-V-pH_12_	496.26	0.29	0.26	1.85	3.65
SSZ-13-V-pH_13_	225.15	0.18	0.09	1.05	2.80
SSZ-13-A-pH_11_	492.59	0.28	0.26	1.16	3.07
SSZ-13-A-pH_12_	503.75	0.30	0.26	1.60	3.29
SSZ-13-A-pH_13_	343.02	0.30	0.12	1.47	2.44
T	327.03	0.26	0.15	2.30	3.08
T-A-pH_12_	355.93	0.27	0.16	2.27	3.11
T-V-pH_12_	376.78	0.28	0.17	2.58	3.38
NaA	—	—	—	2.10	3.13
NaA-A-pH_12_	—	—	—	2.07	3.28
NaA-V-pH_12_	—	—	—	2.13	3.37

a Specific surface area calculated according to the BET model.

b Total pore volume at *P*/*P*_0_ = 0.99.

c Microporous pore volume obtained by the *t*-plot method.

d CO_2_ uptake at 25 °C.

### 3.3 CO_2_ adsorption

**Fig. 4:**
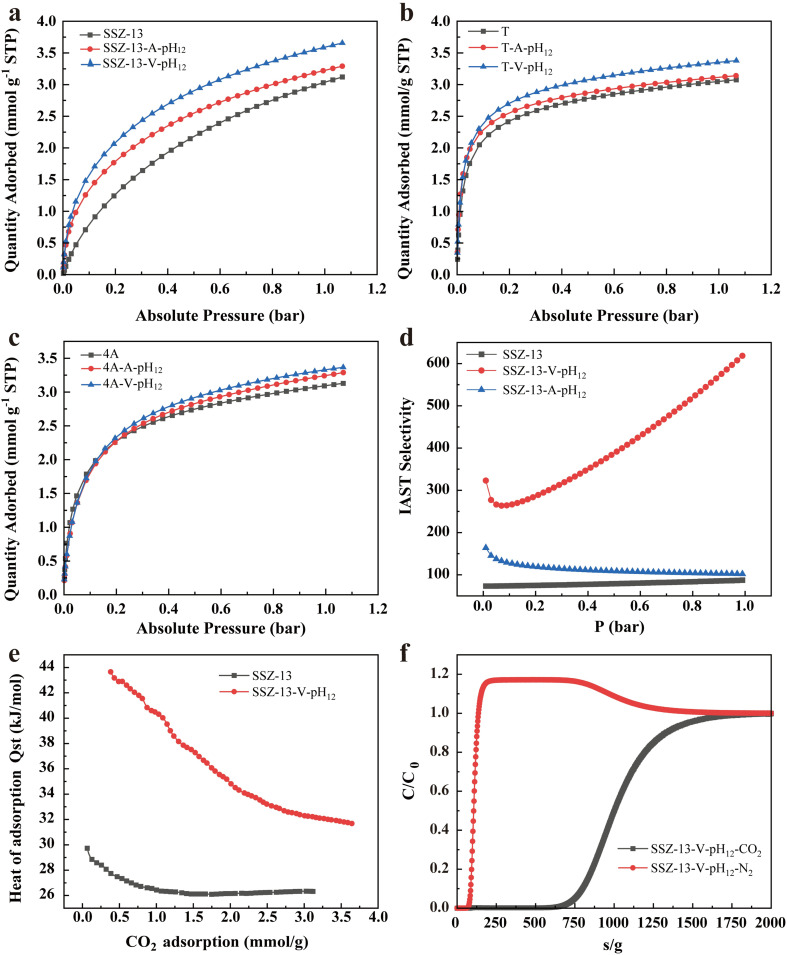
CO_2_ adsorption properties of zeolites. (a–c) CO_2_ adsorption isotherms of SSZ-13 (a), T (b) and NaA (c) before and after alkaline treatment with or without vacuum condition. (d) CO_2_/N_2_ (15/85) IAST selectivity of SSZ-13 before and after alkaline treatment with or without vacuum condition. (e) Heat of adsorption isotherms of SSZ-13 before and after vacuum-assisted alkaline treatment. (f) Breakthrough curve for CO_2_/N_2_ (50/50) mixture at 25 °C.

The CO_2_ adsorption isotherms of SSZ-13, SSZ-13-V-pH_12_ and SSZ-13-A-pH_12_ are illustrated in Fig. 4a. SSZ-13-V-pH_12_ exhibits the highest CO_2_ uptake, reaching 3.65 mmol g^−1^ at 1 bar and 25 °C. This value surpasses that of pristine SSZ-13 by 16.99% and is 10.94% higher than that of SSZ-13-A-pH_12_. At 0.15 bar and 25 °C, the CO_2_ adsorption capacity of SSZ-13-V-pH_12_ is 1.85 mmol g^−1^, showing a notable increase compared to SSZ-13 by 76.19% and SSZ-13-A-pH_12_ by 15.63%. The order in CO_2_ adsorption capacities for SSZ-13, SSZ-13-V-pH_12_, and SSZ-13-A-pH_12_ aligns with that of the H–K micropore volumes of 0.4–0.5 nm aforementioned. In the case of T zeolites (Fig. 4b), T-V-pH_12_ also exhibits a substantial enhancement in CO_2_ adsorption, presenting a 9.7% increase compared to pristine T zeolite. Likewise, NaA-V-pH_12_ displays superior CO_2_ adsorption capacity in comparison to pristine NaA and NaA-A-pH_12_ (Fig. 4c and Table 1). The CO_2_ adsorption capacity of NaA at 1 bar increases from 2.69 mmol g^−1^ to 3.13 mmol g^−1^ after the vacuum-assisted alkaline treatment. These findings clearly demonstrate the remarkable enhancement of CO_2_ adsorption capacity in various zeolites through vacuum-assisted alkaline treatment. In addition, the CO_2_ adsorption capacity for SSZ-13-V-pH_12_, T-V-pH_12_ and NaA-A-pH_12_ are also comparable or somewhat higher than the reported zeolites with same crystal structures (Fig. S3†).

To ensure the reliability of the results, we prepared two additional independent samples for both SSZ-13-A-pH_12_ and SSZ-13-V-pH_12_, The CO_2_ adsorption isotherms are presented in Fig. S4.† The corresponding average adsorption data, including error bars, clearly demonstrate the effectiveness of the vacuum-assisted alkaline treatment. Five consecutive room-temperature cycles without regeneration (Fig. S5†) showed stable performance, with physisorption dominance verifying negligible NaOH residue.

### 3.4 Understanding the enhancement of CO_2_ adsorption

The trend in CO_2_ adsorption ability aligns with that in micropore volume among all the samples (Table 1), suggesting the higher micropore volume contribute to the higher CO_2_ adsorption ability. In addition, beyond the micropore volume, the presence of balanced cations may also impact CO_2_ adsorption through the electrostatic interaction.^24,25^ To delve deeper into this, we analyzed the Na/Al and Si/Al ratios through inductively coupled plasma-optical emission spectrometer (ICP-OES) measurements.^22,26,27^ As shown in Table 2, the Na^+^ content in SSZ-13 apparently increases after the alkaline treatment, indicating the proton substitution by Na^+^. As the pH value increases, the Si/Al ratio of SSZ-13 based system decreases, due to the more significant desilication than dealumination. For T zeolites, the co-participation of K^+^ and Na^+^ in charge balance, combined with slight Al leaching and Si dissolution during alkaline treatment, resulted in a more pronounced variation in the Si/Al ratio for the T-V-pH_12_ sample compared to T-A-pH_12_. The content of Na^+^ doesn't change much after the treatment, but the Na/Al ratio obviously increases, related to the dealumination process. In the case of NaA zeolites, The Si/Al ratio approximates 1, with Na^+^ predominantly dominating the charge compensation. Alkaline treatment exerts minimal compositional impact while maintaining high structural stability. The variations of Na^+^ content, Si/Al and Na/Al ratios are all negligible. We further plot the CO_2_ adsorption capacity against the Na/Al and Si/Al ratio in Fig. S6,† but no significant relationship is found between CO_2_ adsorption capacity and the Si/Al or Na/Al ratio. Thus, the enhancement in CO_2_ adsorption of zeolites *via* vacuum-assisted treatment is mainly attributed to the modified pore size and micropore volumes. To elucidate structural modifications in zeolites, we conducted supplementary FTIR analyses of SSZ-13 samples before and after treatment. As shown in Fig. S7† (normalized spectra), the –OH stretching vibration exhibited significant intensification, while characteristic zeolite bands for Si–O (950–1250 cm^−1^) and Al–O (700–900 cm^−1^) remained unshifted, confirming the superior efficacy of vacuum-assisted alkaline treatment. The absorption band at 3660 cm^−1^ likely originates from defect-associated or extra-framework Al–OH species. Notably, this feature appears most prominent in the ambient-pressure treated sample, indicating greater aluminum leaching under atmospheric conditions that leads to pore blockage through redeposited species.^28–30^

**Table 2:** ICP-OES element analysis of zeolites before and after vacuum-assisted alkaline treatment

Sample	Si [%]	Al [%]	Na [%]	K [%]	Empirical formula	Si/Al	Na/Al
SSZ-13	40.40	4.00	0.07	0.00	Na_0.003_H_3.36_[(AlO_2_)_3.36_(SiO_2_)_32.64_]	9.70	0.02
SSZ-13-A-pH_12_	38.51	3.69	1.51	0.00	Na_0.066_H_3.13_[(AlO_2_)_3.2_(SiO_2_)_32.8_]	10.03	0.48
SSZ-13-V-pH_11_	40.27	3.79	0.281	0.00	Na_0.012_H_3.19_[(AlO_2_)_3.2_(SiO_2_)_32.8_]	10.21	0.09
SSZ-13-V-pH_12_	40.63	4.34	2.13	0.00	Na_0.093_H_3.51_[(AlO_2_)_3.6_(SiO_2_)_32.4_]	8.99	0.58
SSZ-13-V-pH_13_	34.29	4.67	5.28	0.00	Na_0.229_H_4.07_[(AlO_2_)_4.3_(SiO_2_)_31.7_]	7.05	1.33
T	37.19	10.74	3.43	7.37	Na_0.149_K_0.188_H_7.96_[(AlO_2_)_8.3_(SiO_2_)_27.7_]	3.33	0.37
T-A-pH_12_	30.93	9.08	3.41	6.36	Na_0.148_K_0.163_H_7.79_[(AlO_2_)_8.1_(SiO_2_)_27.9_]	3.27	0.44
T-V-pH_12_	29.92	8.69	3.54	6.23	Na_0.154_K_0.159_H_7.69_[(AlO_2_)_8.0_(SiO_2_)_28.0_]	3.31	0.48
NaA	17.87	16.33	13.80	0.00	Na_0.600_[(AlO_2_)_11.7_(SiO_2_)_12.3_]	1.05	0.99
NaA-A-pH_12_	18.03	17.76	13.85	0.00	Na_0.602_[(AlO_2_)_12.1_(SiO_2_)_11.9_]	0.98	0.92
NaA-V-pH_12_	16.44	16.17	12.96	0.00	Na_0.564_[(AlO_2_)_12.0_(SiO_2_)_12.0_]	0.98	0.94

The exceptional CO_2_ adsorption performance of SSZ-13-V-pH_12_ prompted a detailed evaluation of the CO_2_ adsorption heat and kinetics. According to the Clapeyron–Clausius equation with the adsorption data at 273 K and 298 K (Fig. S8†),^3,31^ CO_2_ adsorption heat of SSZ-13 is calculated, as shown in Fig. 4d. The CO_2_ adsorption heat for the pristine SSZ-13 ranges from 26 to 30 kJ mol^−1^. For SSZ-13-V-pH_12_ sample, it increases to 32–44 kJ mol^−1^, corresponding to stronger interaction between SSZ-13-V-pH_12_ and CO_2_. Moreover, fast CO_2_ adsorption kinetics is desirable for practical applications. Adsorbents with high CO_2_ adsorption capacity often exhibit high adsorption heat but relatively slow adsorption kinetics, due to the poor accessibility of adsorption sites and/or too strong affinity toward CO_2_.^32–34^ As shown in Fig. S9,† the CO_2_ adsorption kinetics of SSZ-13-V-pH_12_ is quite fast (<50 s to reach maximum) and comparable to that of the pristine SSZ-13, suggesting that the enhancement in CO_2_ adsorption does not compromise the fast CO_2_ adsorption kinetics. The CO_2_/N_2_ selectivity is further evaluated using the ideal adsorbed solution theory (IAST).^35^ The N_2_ adsorption data used for the IAST calculation are presented in Fig. S10.† As displayed in Fig. 4d, the IAST selectivity of SSZ-13-V-pH_12_ is nearly one order of magnitude higher than that of pristine SSZ-13, which can be attributed to the Na^+^ enrichment. Similarly, the selectivity of T or NaA zeolite is improved as well (Fig. S11†). Using a dynamic gas breakthrough apparatus, breakthrough experiments are further performed to examine the competitive adsorption of CO_2_ and N_2_.^36^ As shown in Fig. 4f, N_2_ elutes rapidly from the sample column, whereas CO_2_ exhibits significantly delayed elution. The extrapolated CO_2_ adsorption capacity of SSZ-13-V-pH_12_ is 2.95 mmol g^−1^ at 0.5 bar, almost the same as the single component CO_2_ adsorption capacity (2.90 mmol g^−1^).

## 4. Conclusion

In conclusion, a universal method is proposed to augment the CO_2_ adsorption capacities of zeolites, namely the vacuum-assisted alkaline treatment. According to extensive investigations involving pore size distribution analysis, variation in Si/Al ratio, and effect of surface cation, the improved CO_2_ adsorption is mainly attributed to the alteration in pore size and micropore volume, caused by desilication and cation exchange during the vacuum-assisted alkaline treatment. The utilization of vacuum conditions helps the release of guest molecules trapped in the micropores, facilitating the penetration of the solution during alkaline treatment. Overall, this study provides a straightforward and scalable strategy to modulate the inner surface of porous materials.

## Conflicts of interest

There are no conflicts to declare.

## Supplementary Material

RA-015-D5RA01559F-s001

## Data Availability

The data supporting this article have been included as part of the ESI.†
