# Supplementary material for: Facile method for enhancing the CO2 adsorption capacity of zeolites through vacuum-assisted alkaline treatment
Source: RSC Adv. 2025 Jun 11;15(25):19802–9. doi: 10.1039/d5ra01559f (PMC12152726; doi:10.1039/d5ra01559f)
Supplement: RA-015-D5RA01559F-s001 [file RA-015-D5RA01559F-s001.pdf]

Supplementary Information (SI) for RSC Advances.
This journal is © The Royal Society of Chemistry 2025

Supplementary Information：

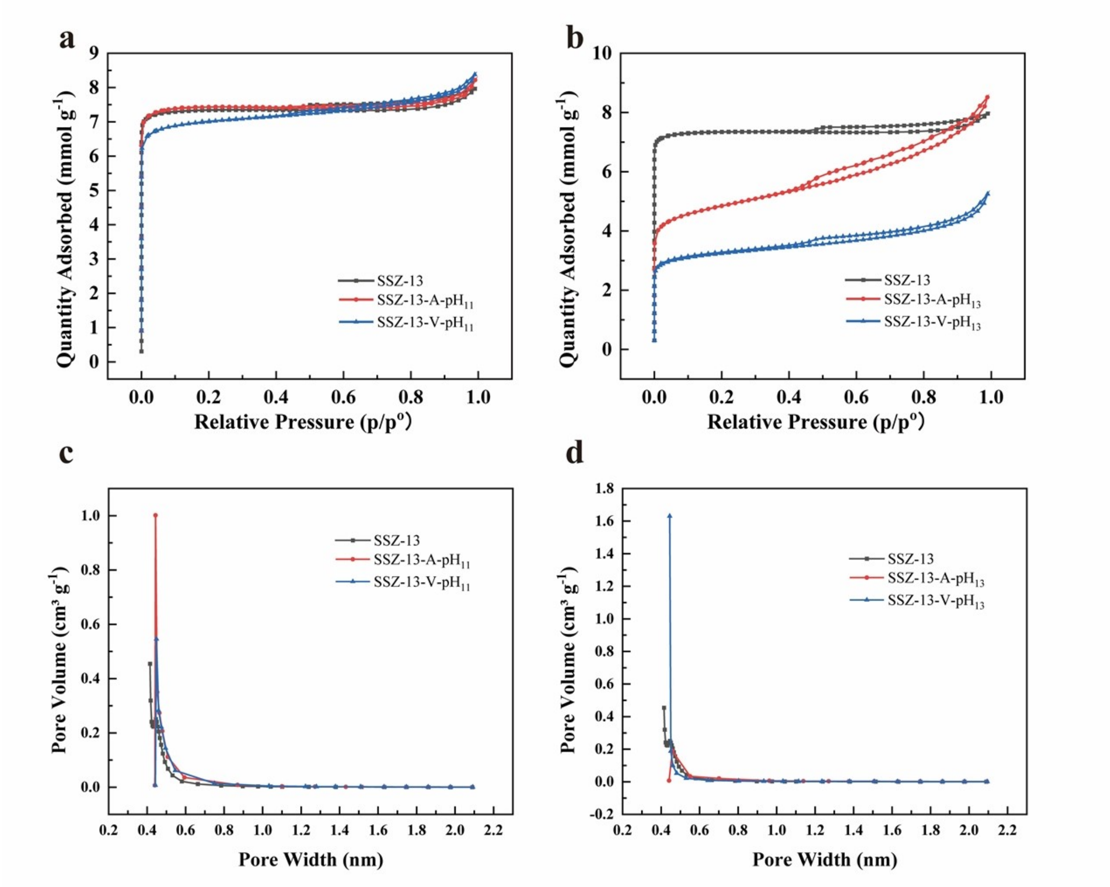

Fig. S1. 77 K Nitrogen adsorption/desorption curves and H-K pore size distribution profiles of SSZ-13 treated at different pH. (a, c) pH=11; (b, d) pH=13.

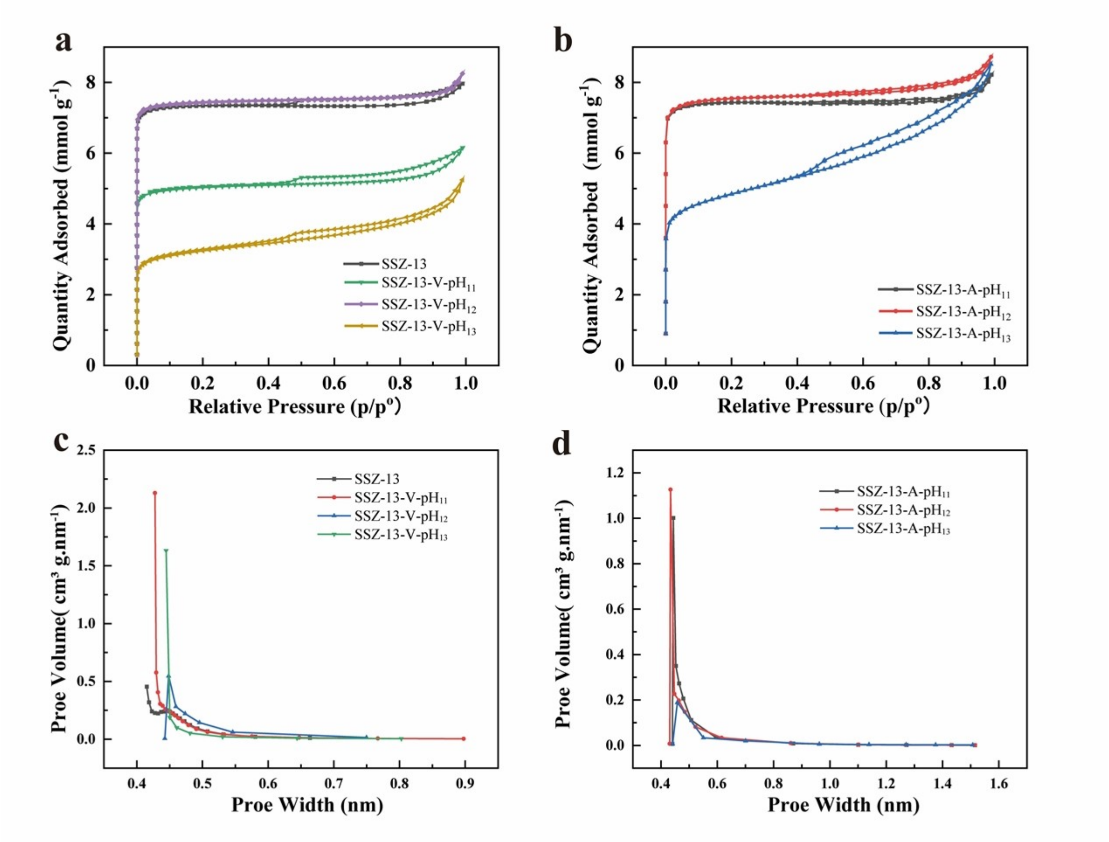

Fig. S2. 77 K N_2 adsorption/desorption isotherms of SSZ-13 and the corresponding pore size distribution curves. (a) 77 K N_2 adsorption/desorption isotherms for SSZ-13, SSZ-13-V-pH_{11}, SSZ-13-V-pH_{12} and SSZ-13-V-pH_{13}; (b) 77 K N_2 adsorption/desorption isotherms for SSZ-13-A-pH_{11}, SSZ-13-A-pH_{12} and SSZ-13-A-pH_{13}; (c) H-K pore size distribution curves of SSZ-13, SSZ-13-V-pH_{11}, SSZ-13-V-pH_{12} and SSZ-13-V-pH_{13}; (d) H-K pore size distribution curves of SSZ-13-A-pH_{11}, SSZ-13-A-pH_{12} and SSZ-13-A-pH_{13}.

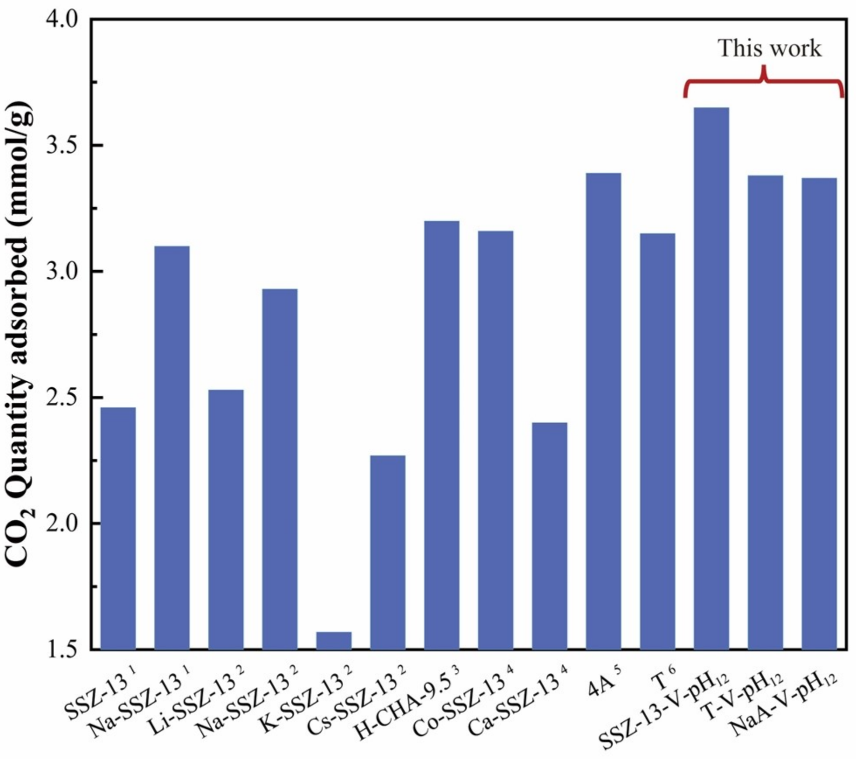

Fig. S3. Comparison of CO_2 adsorption properties between the zeolites synthesized in this study and other reported zeolites with the same crystal structures.[2-7]

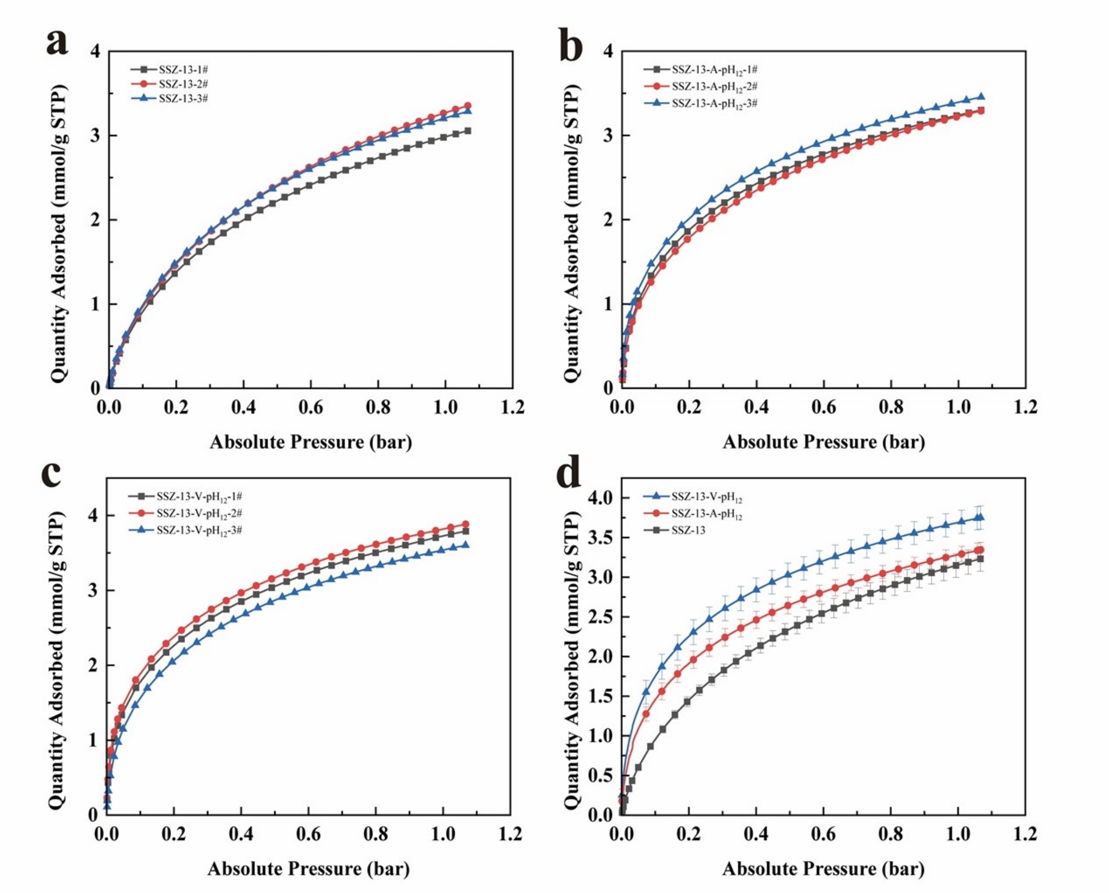

Fig. S4. Independent samples of SSZ-13 before and after (vacuum-assisted) alkaline treatments. (a) SSZ-13; (b) SSZ-13-A-pH_{12}; (c) SSZ-13-V-pH_{12}; (d) The average data with error bars.

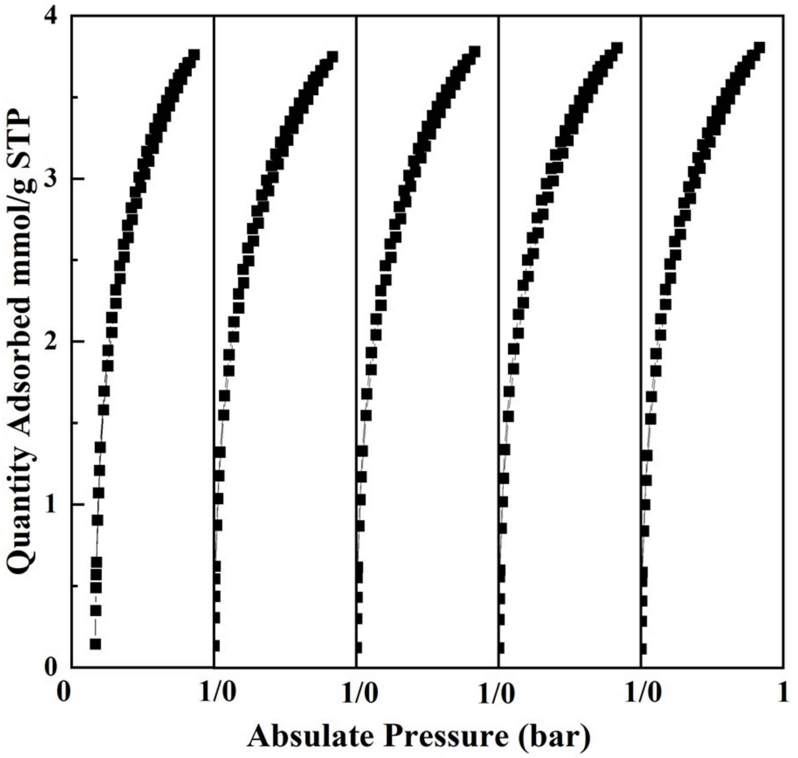

Fig. S5. Cyclic CO_2 adsorption isotherms of SSZ-13-V-pH_{12} at 25°C.

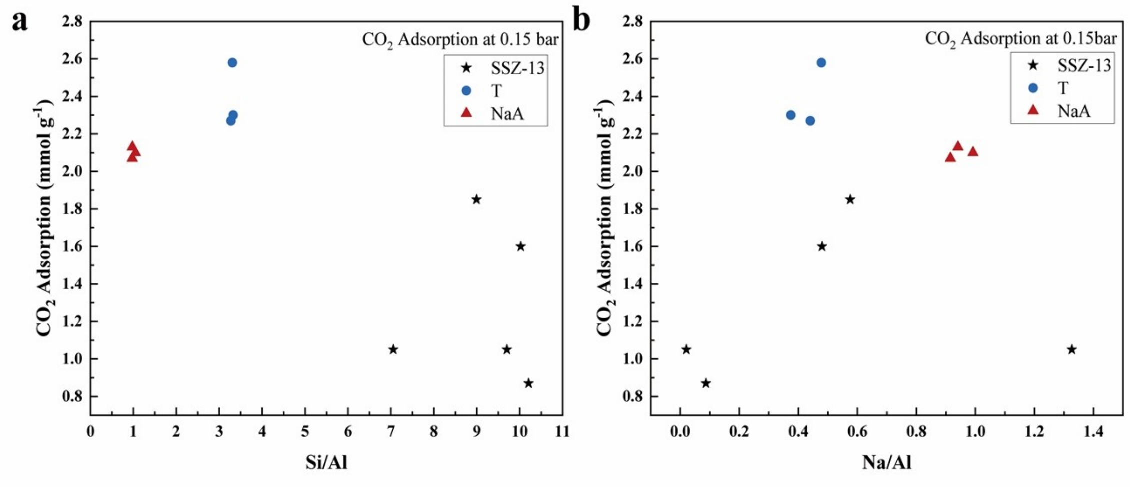

Fig. S6. CO_2 adsorption capacity of zeolites against the Si/Al ratio (a) and the Na/Al ratio (b).

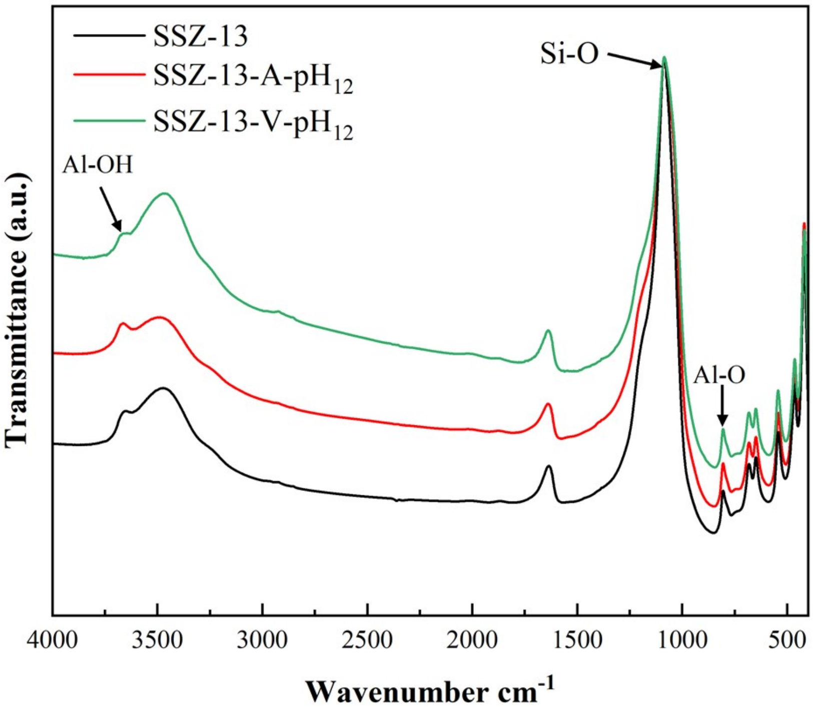

Fig. S7. FTIR curves of SSZ-13, SSZ-13-A-pH_{12} and SSZ-13-A-pH_{12}.

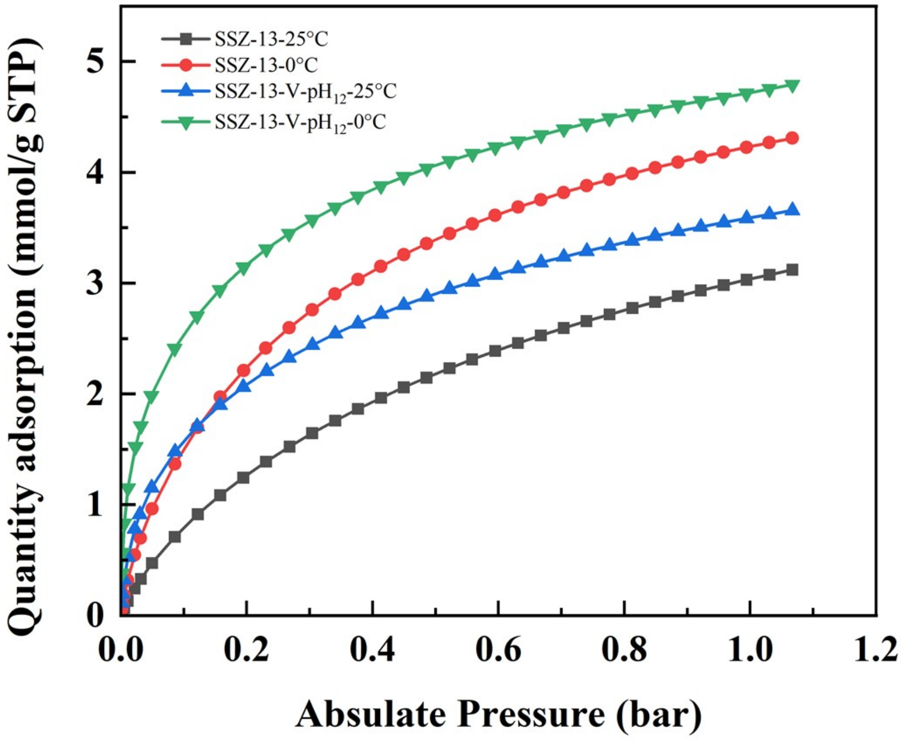

Fig. S8. CO_2 adsorption isotherms of SSZ-13 before and after vacuum-assisted alkaline treatment at 0°C and 25°C.

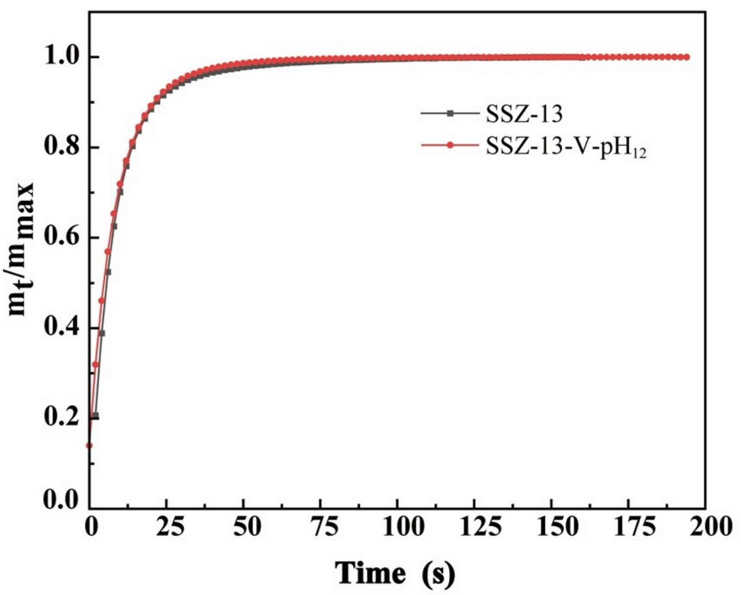

Fig. S9. Kinetic CO_2 adsorption curves of SSZ-13 before and after vacuum-assisted alkaline treatment.

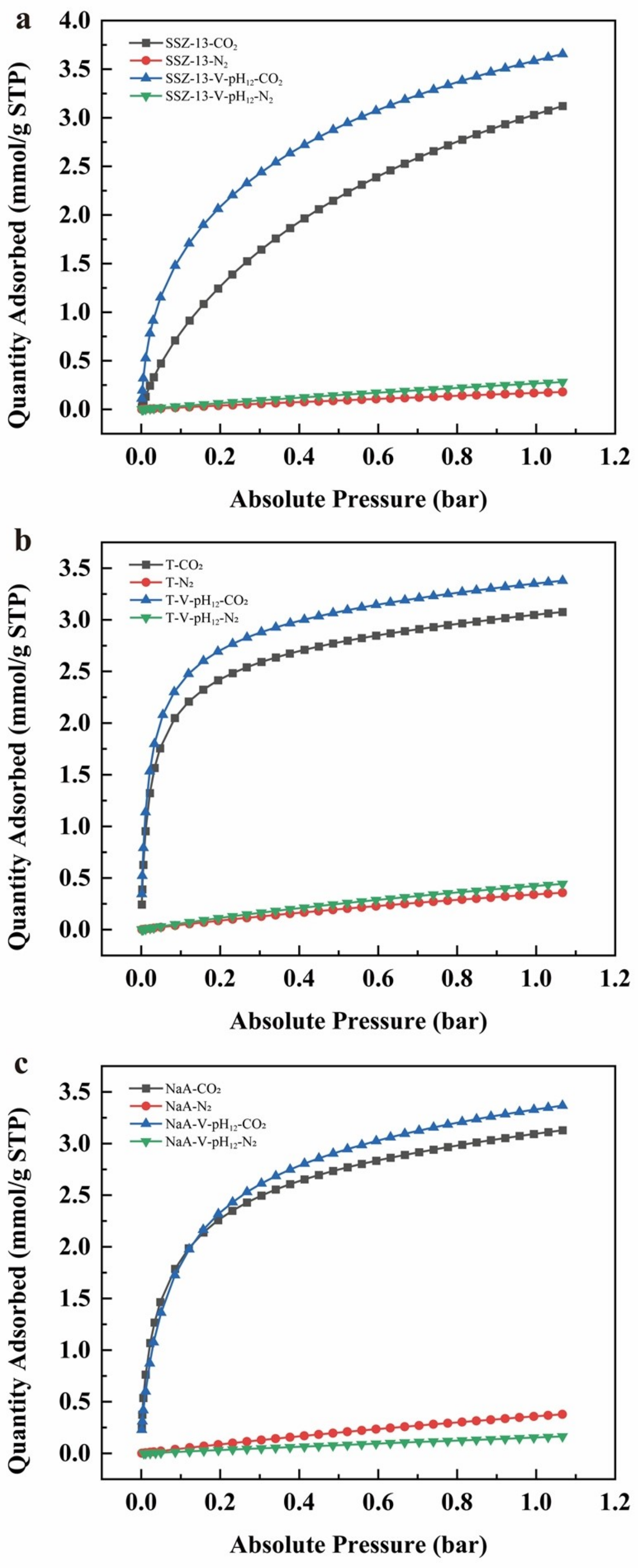

Fig. S10. CO_2 and N_2 adsorption isotherms of molecular sieve before and after vacuum-assisted alkaline treatment at 25°C. (a) SSZ-13; (b) T zeolites; (c) NaA molecular sieve.

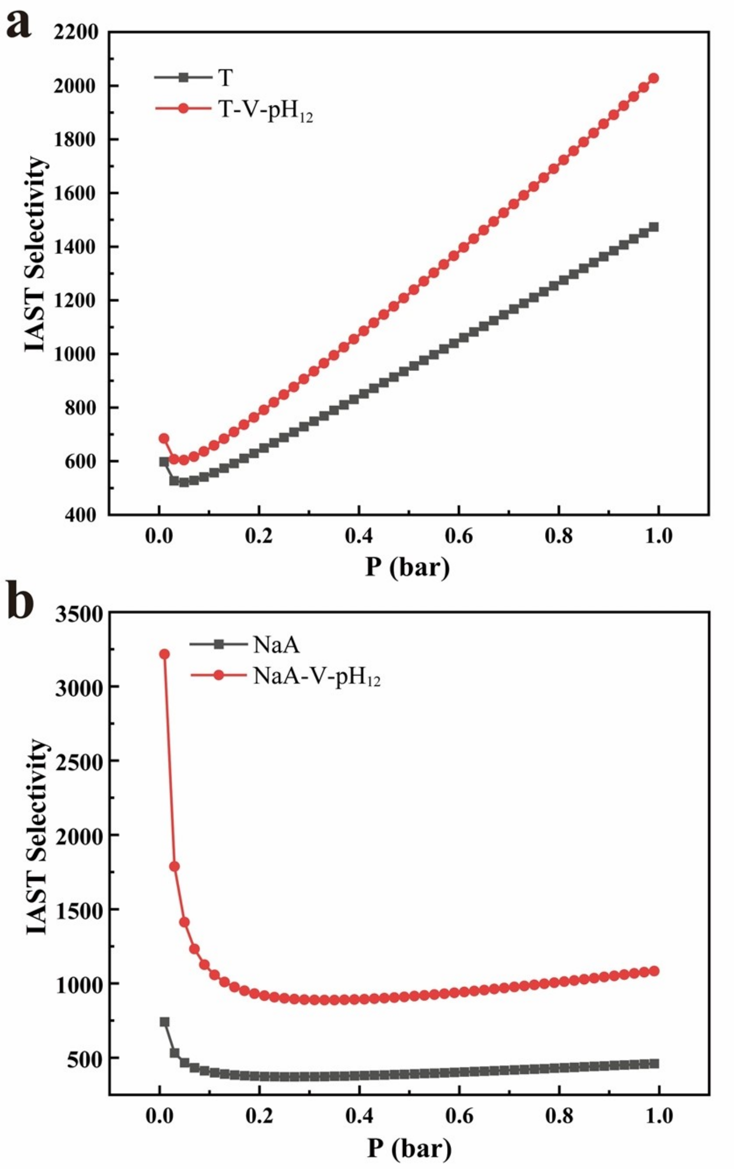

Fig. S11. IAST-predicted CO_2/N_2 selectivity with at 25 °C. (a) T zeolites; (b) NaA molecular sieve.

Table S1. Summary of data from X-Ray Fluorescence (XRF) analysis.

Sample	Si [%]	Al [%]	Na [%]	K [%]	Si/Al
SSZ-13	42.98	4.21	-	-	9.81
T	32.33	9.43	3.21	7.17	3.29
4A	19.33	19.08	16.60	-	0.97

References

1. M. R. Hudson, W. L. Queen, J. A. Mason, D. W. Fickel, R. F. Lobo and C. M. Brown, *J. Am. Chem. Soc.*, 2012, **134**, 1970-1973.
2. Y. L. Jiang, W. Zhou, N. He, S. Y. Yan, S. Y. Chen and J. X. Liu, *Chemcatchem*, 2022, **14**.
3. J. W. Feng, Y. F. Hu, Q. Bao, D. Liang and Y. Xu, *Micro Nano Lett.*, 2020, **15**, 529-534.
4. Y. Guo, T. J. Sun, Y. M. Gu, X. W. Liu, Q. L. Ke, X. L. Wei and S. D. Wang, *Chem-Asian J.*, 2018, **13**, 3222-3230.
5. D. X. Gai, Y. X. Guan, D. Ma, Y. Y. Deng, J. C. Dong, Z. Y. Wang, J. L. Li, I. Agirrezabal-Telleria and X. Q. Zou, *J. Membrane. Sci.*, 2025, **722**.
6. D. Panda, E. A. Kumar and S. K. Singh, *Ind. Eng. Chem. Res.*, 2019, **58**, 5301-5313.
7. M. D. Rad, S. Fatemi and S. M. Mirfendereski, *Chem. Eng. Res. Des.*, 2012, **90**, 1687-1695.
